# Implementation of zero or near-zero fluoroscopy catheter ablation for idiopathic ventricular arrhythmia originating from the aortic sinus cusp

**DOI:** 10.1007/s10554-021-02432-8

**Published:** 2021-10-28

**Authors:** Katarzyna Styczkiewicz, Bartosz Ludwik, Marek Styczkiewicz, Janusz Śledź, Małgorzata Gorski, Sebastian Stec

**Affiliations:** 1 Department of Internal Medicine, Institute of Medical Sciences, Medical College, University of Rzeszów, Al. mjr.W.Kopisto 2a, 35-310 Rzeszów, Poland; 2 Department of Cardiology, Centre for Research and Development, Regional Specialist Hospital, Wrocław, Poland; 3 Department of Cardiology, The Pope John Paul II Province Hospital of Zamość, Zamość, Poland; 4 ELMedica, EP-Network, Skarzysko-Kamienna, Poland; 5 Department of Cardiology, Specialist Hospital in Wałbrzych, Wałbrzych, Poland

**Keywords:** Catheter ablation, Aortic cusp, Idiopathic ventricular arrhythmia, Fluoroscopy, Electroanatomic mapping

## Abstract

Complete elimination of fluoroscopy during radiofrequency ablation (RFA) of idiopathic ventricular arrhythmias (IVAs) originating from the aortic sinus cusp (ASC) is challenging. The aim was to assess the feasibility, safety and a learning curve for a zero-fluoroscopy (ZF) approach in centers using near-zero fluoroscopy (NOX) approach in IVA-ASC. Between 2012 and 2018, we retrospectively enrolled 104 IVA-ASC patients referred for ZF RFA or NOX using a 3-dimensional electroanatomic (3D-EAM) system (Ensite, Velocity, Abbott, USA). Acute, short and long-term outcomes and learning curve for the ZF were evaluated. ZF was completed in 62 of 75 cases (83%) and NOX in 32 of 32 cases (100%). In 13 cases ZF was changed to NOX. No significant differences were found in success rates between ZF and NOX, no major complications were noted. The median procedure and fluoroscopy times were 65.0 [45–81] and 0.0 [0–5] min respectively, being shorter for ZF than for NOX. With growing experience, the preference for ZF significantly increased—43% (23/54) in 2012–2016 vs 98% (52/53) in 2017–2018, with a simultaneous reduction in the procedure time. ZF ablation can be completed in almost all patients with IVA-ASC by operators with previous experience in the NOX approach, and after appropriate training, it was a preferred ablation technique. The ZF approach for IVA-ASC guided by 3D-EAM has a similar feasibility, safety, and effectiveness to the NOX approach.

**Supplementary Information:**

The online version contains supplementary material available at 10.1007/s10554-021-02432-8.

## Introduction

Radiofrequency ablation (RFA) of idiopathic ventricular arrhythmia (IVA) originating from the aortic sinus cusp (ASC) is an effective treatment method, although it is challenging due to the potential risk of collateral damage and severe complications [1–7]. Available data show that catheter ablation of IVA-ASC can be safely performed [1–5], although at present, it is usually guided by fluoroscopy or modern imaging modalities such as intracardiac echocardiography. Zero-fluoroscopy (ZF) mapping and navigation with 3-dimensional electroanatomical (3D-EAM) mapping systems initially allowed safe non-fluoroscopic guidance in pediatric patients and pregnant women [8, 9], in whom X-ray exposure should be particularly avoided. However, recent evidence has indicated that non-fluoroscopic ablation of IVA, mostly right-sided, is feasible and safe also in other adult populations [10, 11]. However, there are scarce data on a ZF approach in patients with IVA-ASC [12, 13]. Therefore, the aim of this retrospective multicenter standardized registry study was to assess the implementation, feasibility, learning curve, safety, and efficacy of the ZF approach in centers using a near-zero fluoroscopy (NOX) approach for RFA of idiopathic premature ventricular complexes (PVCs) or ventricular tachycardias (VTs) originating from the ASC.

## Materials and methods

### Patient population

The study population included patients from the retrospective observational multicenter “Electro” registry with reviewing collected data (RARE-A-CAREgistry-REGISTRY of RARE Arrhythmias and their Comprehensive oR Atypical managemEnt). The registry covered ablation procedures from 10 Polish electrophysiology (EP) centers performed by the same team of 3 experienced operators and 3 middle-advanced EP fellows in training. Patients undergoing RFA for frequent idiopathic PVCs and/or VTs originating from the ASC were enrolled from January 2012 to August 2018. They were unselected and referred for ablation with either a ZF or NOX approach using the Ensite Velocity NavX system (Abbott, St. Paul, Minnesota, United States) without support of intracardiac or transesophageal echocardiography. The choice of the approach was based on the experience of the first operator. It was required before using ZF for aortic arch arrhythmias due to a predefined training approach. In this registry, three experienced operators and three middle-advanced fellows performed ZF as an intention-to-treat approach in each case when they started procedure without lead-apron. If the operators performed more than 100 ZF procedures for other indications (supraventricular regular arrhythmias, pre-excitation and right-sided idiopathic PVC/VT) and at least 50 procedures with a retrograde transaortic approach, ZF approach was chosen as a primary approach. All ablation procedures were done in symptomatic patients in line with current guidelines [4] after failure of medical treatment or directly in shared-decision making process between patient, referring physician and electrophysiologist.

### Mapping and ablation protocol

The ablation was performed according to a previously published protocol [14]. As described above, two techniques were used: ZF or NOX. The NOX approach uses the ALARA principles (As Low As Reasonably Achievable) and a 2-catheter approach (1 decapolar diagnostic catheter and 1 ablation catheter). Fluoroscopy at a rate of 4–6 frames per second was used. In the ZF approach, the fluoroscopy arm was kept far away from the patient table and laboratory staff did not wear lead protection, which was only used when switching to radiation was required. Using the 3D-EAM system, intracardiac signals and consistent and reproductive impedance rise (an increase in impedance above 20–30 Ohms) and typical catheter movement and stability within coronary ostia, left main ostium, or occassionally right coronary artery ostium, were mapped to assess the appropiate distance of the target site from the coronary artery ostia.

**Fig. 1:**
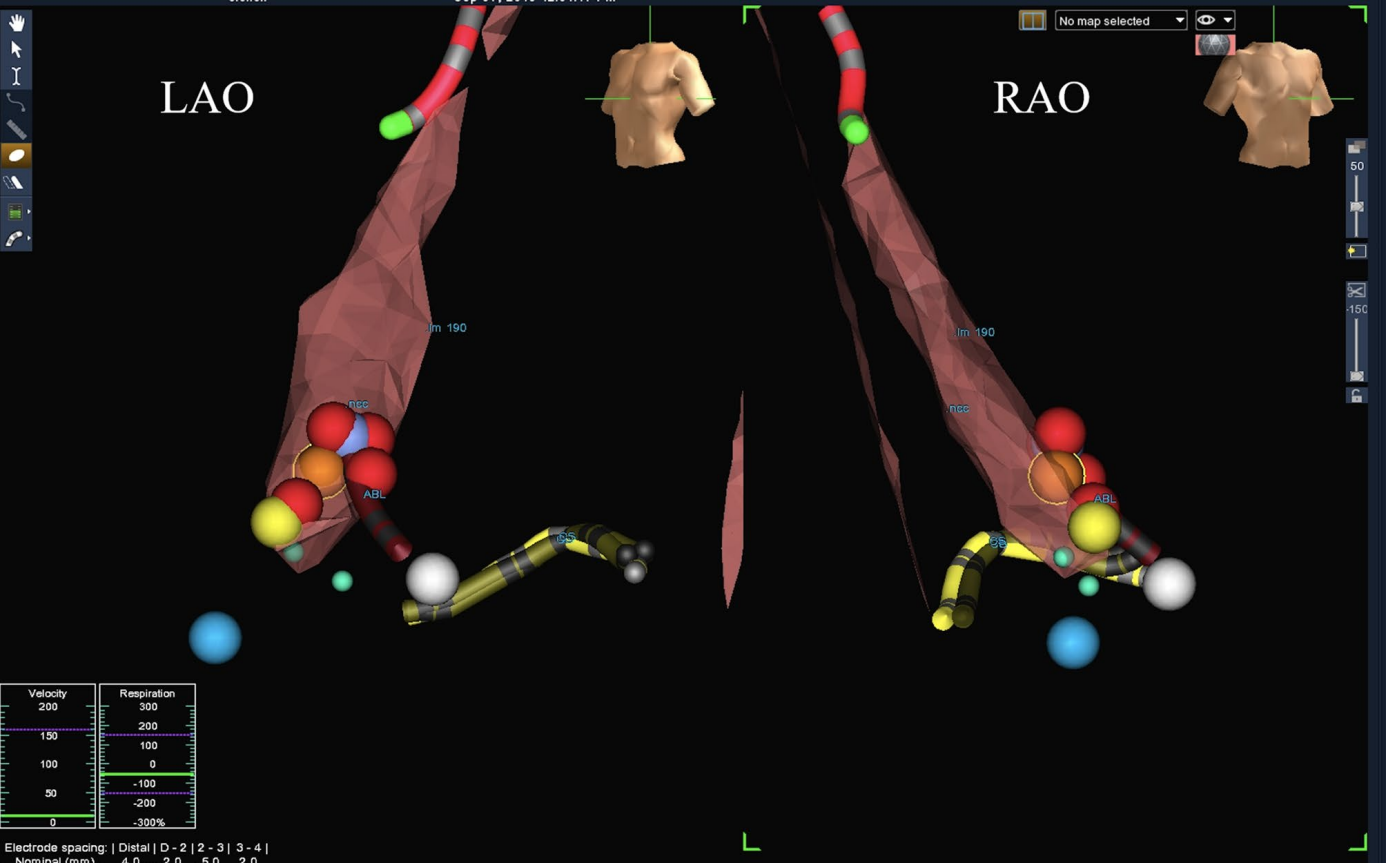
Example of transaortic, retrograde, 3-dimensional electroanatomic mapping in zero-fluoroscopy catheter ablation of premature ventricular complex/ventricular tachycardia originating from the junction of the left and right coronary cusps. Left (LAO) and right anterior oblique (RAO) biplane view. LM 190 denotes left main ostium (an increase in impedance to 190 Ohms as compared with the mean impedance of 140 Ohms in the aortic root). Blue balls represent the recording of the His bundle potential from the right side, green balls represent the recording of the left far-field His bundle potential, yellow balls represent the clearest recording of the His bundle potential from the cusp marked to avoid ablation in this area, red balls represent the ablation site, and finally, violet ball represents the final effective ablation site. Pink contour represents the aortic root, and yellow shadow, a decapolar catheter located in the coronary sinus. *ABL* tip of ablation catheter, *ncc* non coronary cusp, *CS* coronary sinus, *lm 190* left main artery ostium. (Color figure online)

Moreover, simple electroanatomic mapping was performed to visualize the ASC and His bundle signals (Fig. 1). After 10 cases of monitored procedures by expert, operators were encouraged to use ZF mapping of the coronary ostia and aortic root as the preferred approach. Appropriate and accurate electroanatomical mapping as compared to coronary angiography was required to proceed to ZF approach. Gathering experience with ZF approach in hundreds of procedures, especially with retrograde approach, resulted in appropriate validation of electroanatomic maps of aortic cusps as well as impedance rise, difference and catheter position from left main and right coronary artery ostium. Therefore, pure electroanatomic mapping were sufficient to validate position of the tip of ablation catheter before RF application.

The procedure time was defined as the time from the first femoral puncture to catheter removal at the end of the RFA procedure. Fluoroscopy time was defined as the total duration of fluoroscopy during the whole RFA procedure.

A successful outcome of the RFA procedure was defined as complete disappearance of target PVC or VT morphology for more than 15 min after the last application, PVC/VT non-inducibility after isoproterenol infusion and complete electrophysiological study using incremental atrial pacing, as well as up to 3 programmed ventricular and atrial extrastimuli. The PVC/VT origin was classified as left coronary cusp (LCC), right coronary cusp (RCC), LCC/RCC junction (LCC/RCC), noncoronary cusp (NCC), or aortomitral continuity (AMC)/LCC area (when both sites were required for a successful ablation).

### Follow-up assessment

The periprocedural (acute), short-term (1 month after RFA), and long-term (12 months after RFA) outcomes, as well as the learning curve for using ZF in RFA for IVA-ASC, were evaluated, and the reasons for switching from the ZF to NOX approach were obligatory required and reported in procedural reports. Data for all patients were obtained using a standardized data collection form, including demographic characteristics, details on arrhythmia, procedure-related complications, and details on the RFA procedure (total procedure time, fluoroscopy time, and RFA delivery time). A standard definition of major complications was used (Supplementary Table S1). Treatment success was defined as a post-ablation decrease of 80% or more in the number of PVC/VT on 24-h Holter monitoring performed before ablation and repeated at 1 and 12 months. Transthoracic echocardiography was performed before RFA and at 1 and 12 months.

### Statistical analysis

Categorical variables were described using counts and percentages. Quantitative variables were described using medians and interquartile ranges (Q1–Q3). The null hypothesis was tested using the Mann–Whitney test or *t*-test. For categorical variables, significant differences between groups were assessed using the Chi-square or Fisher exact tests. A *P* value of less than 0.05 indicated statistical significance. No adjustment for multiple comparisons was made. All statistical analyses were performed using R 3.4 (R Foundation for Statistical Computing, Vienna, Austria).

## Results

### Baseline characteristics of patients

Between January 2012 and August 2018, we enrolled 104 consecutive patients undergoing RFA due to IVA-ASC. The median age of the study group was 55.0 [mean (SD): 49.5 (19.7); range: 10.0–86.0, Q1–Q3, 33–64] years. There were 53 men (51%) and 7 children (7%) under the age of 18 years. Echocardiography at baseline showed the median left ventricular ejection fraction of 60.0% [Q1–Q3 55.0–64.0%], and 24-h Holter monitoring revealed the median number of PVCs of 18,102.5 [Q1–Q3, 10,925.2–30,148.8] (Table 1). Among the 107 PVCs from the ASC, the main sites of origin were the LCC (n = 49, ZF-28, NOX-21), AMC/LCC area (n = 25, ZF-22, NOX-3), and LCC/RCC junction (n = 21, ZF-17, NOX-4). The less frequent locations were the RCC (n = 6, ZF-4, NOX-2) and NCC (n = 6, ZF-4, NOX-2).

**Table 1:** Baseline characteristics of patients by the radiofrequency catheter ablation approach

	ZF approach	NOX approach	*P*
Mean age, years	56.0 [31.5–67.0]	53.0 [33.0–61.0]	0.11
BMI, kg/m^2^	26.4 [23.8–30.4]	28.7 [23.4–32.0]	0.24
Male sex	37 (49.3%)	17 (53.1%)	0.88
LVEF before ablation, %	60.0 [55.0–64.2]	56.5 [50.0–63.0]	0.22
Number of PVCs before ablation	17290.0 [9938.0–30000.0]	20089.0 [15036.5–30216.5]	0.34

Continuous variables are expressed as medians (Q1–Q3)

Categorical variables are expressed as number (%) of patients

*BMI* body mass index, *LVEF* left ventricular ejection fraction, *NOX* near-zero fluoroscopy, *PVC* premature ventricular complex, *ZF* zero-fluoroscopy

### Procedural details and ablation outcome

An intention-to-treat analysis showed that RFA with the ZF approach was completed in 62 of the 75 cases (82.7%), and with the NOX approach, in 32 of the 32 cases (100%). The baseline characteristics of patients by the approach used are shown in Table 1.

**Fig. 2:**
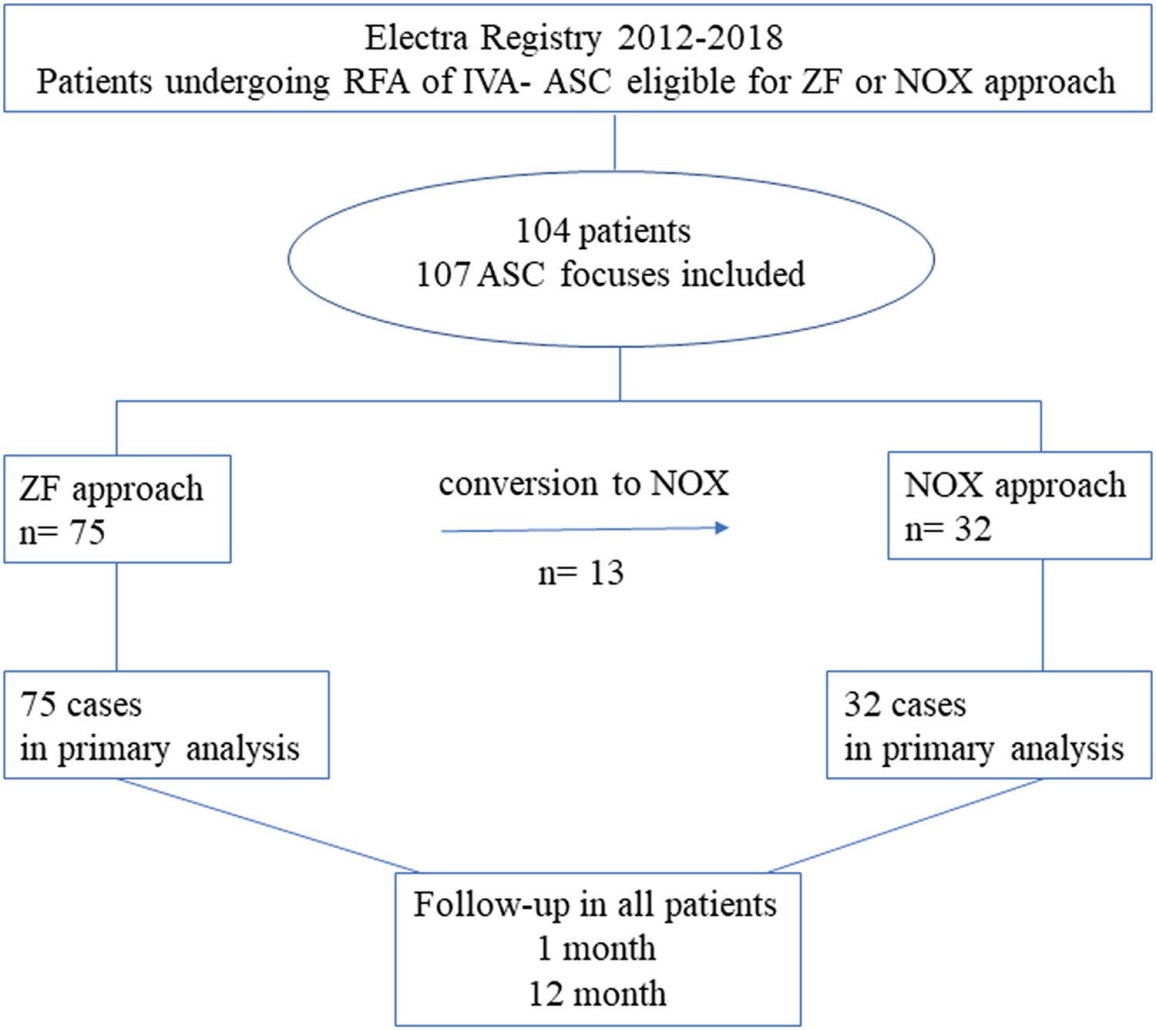
Flowchart of patient enrollment to the study and follow-up. *ASC* aortic sinus cusp, *IVA* idiopathic ventricular arrhythmia, *NOX* near-zero fluoroscopy, *RFA* radiofrequency ablation, *ZF* zero fluoroscopy

The reasons for switching from ZF to NOX included the need for elective aortography (the first operator choice) combined with coronary angiography (n = 6), urgent coronary angiography due to chest pain and ST-segment deviation related to transient coronary spasms during mapping prior to ablation (n = 2), assessment of catheter stability (n = 3), confirmation of the femoral access site (n = 1), and navigation problem (n = 1). The flowchart of patient enrollment to the study is shown in Fig. 2.

The median total procedure time was 65.0 [range 45.0–81.0] min, fluoroscopy time was 0.0 [range 0.0–5.0] min, and RFA delivery time was 5.8 [range 3.3–9.2] min. The procedural details with respect to the RFA approach are shown in Table 2. The median procedure time and fluoroscopy time were significantly lower for the ZF compared with NOX approach. Irrigated catheters were used in 42 (40%) of patients based on physician choice.

**Table 2:** Procedural parameters in patients undergoing radiofrequency catheter ablation with zero fluoroscopy or near-zero fluoroscopy approach for idiopathic ventricular arrhythmias originating from the aortic sinus cusp

Parameter	ZF approach	NOX approach	*P*
Procedure time, min	58.0 [45.0–75.0]	75.0 [65.0–89.8]	0.002
Fluoroscopy time, min	0.0 [0.0–0.0]	6.0 [2.0–11.8]	< 0.001
RFA delivery time, min	6.2 [3.2–8.8]	5.1 [3.4–11.3]	0.93

Continuous variables are expressed as medians [Q1–Q3]

*NOX* near-zero fluoroscopy, *RFA* radiofrequency ablation, *ZF* zero-fluoroscopy

**Fig. 3:**
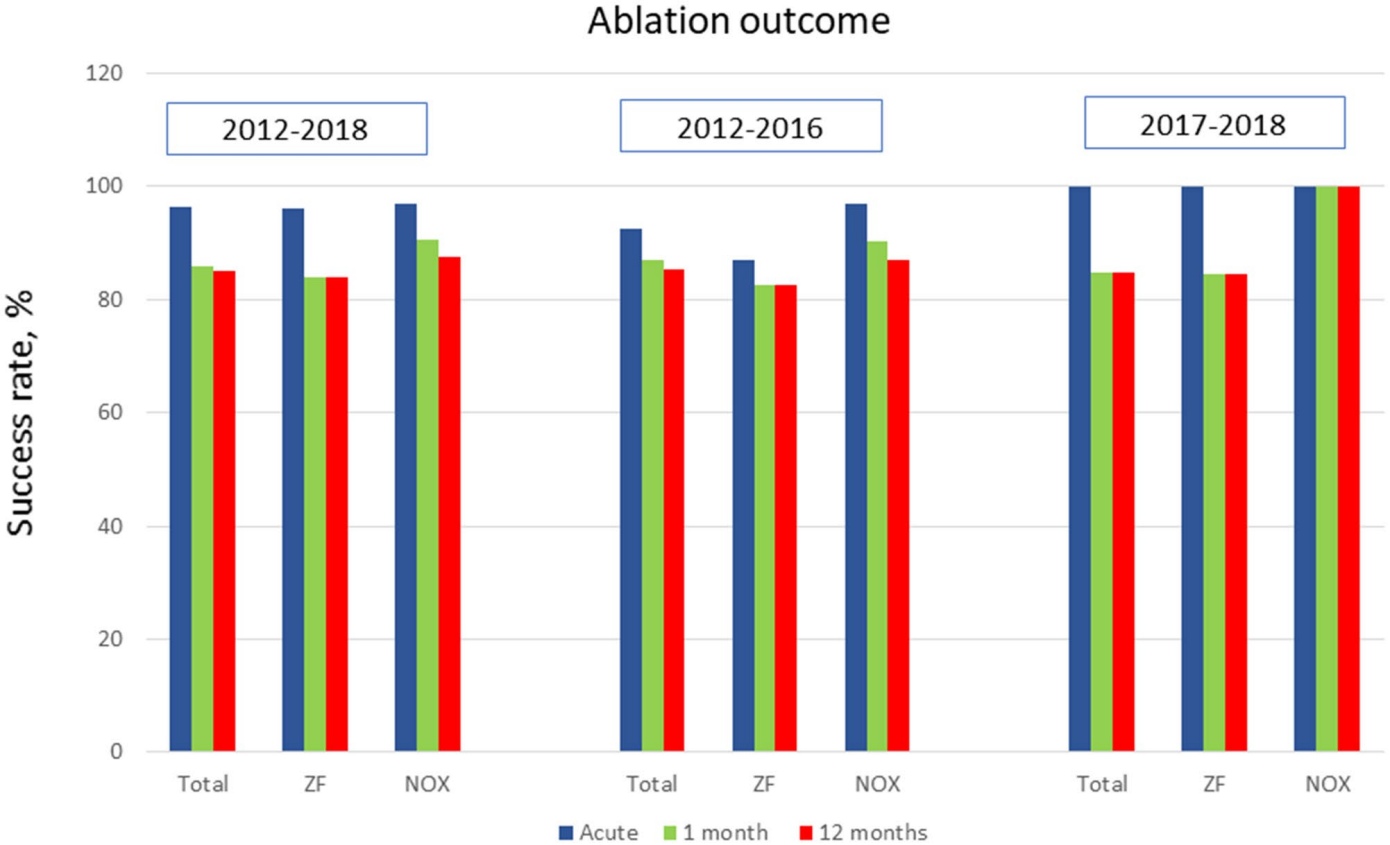
Ablation outcome of ablation with zero fluoroscopy or near-zero fluoroscopy for idiopathic ventricular arrhythmias from the aortic sinus cusp in the early (2012–2016) and late study period (2017–2018). The groups did not differ in success rates considering periprocedural, 1-month, and 12-month follow-up. *NOX* near-zero fluoroscopy, *ZF* zero fluoroscopy

The periprocedural, short-term, and long-term success rates were 96%, 86%, and 85%, respectively. No significant differences in periprocedural, 1-month, and 12-month outcomes of ablation were found between the ZF and NOX approaches (Fig. 3). During the follow-up, 4% of patients underwent repeat ablation procedures due to IVA recurrence or procedural failure.

No major complications (listed in Supplementary Table S1) related to the procedures were noted in any of the groups. Minor complications included 2 patients with a transient short-lasting uncomplicated coronary spasm during the ZF procedure (one before RF application, the other after initiating RF applications). However, they had normal coronary vessels on coronary angiography performed after ablation, and acute myocardial infarction was excluded.

### Operator experience and the learning curve for the zero-fluoroscopy approach

**Fig. 4:**
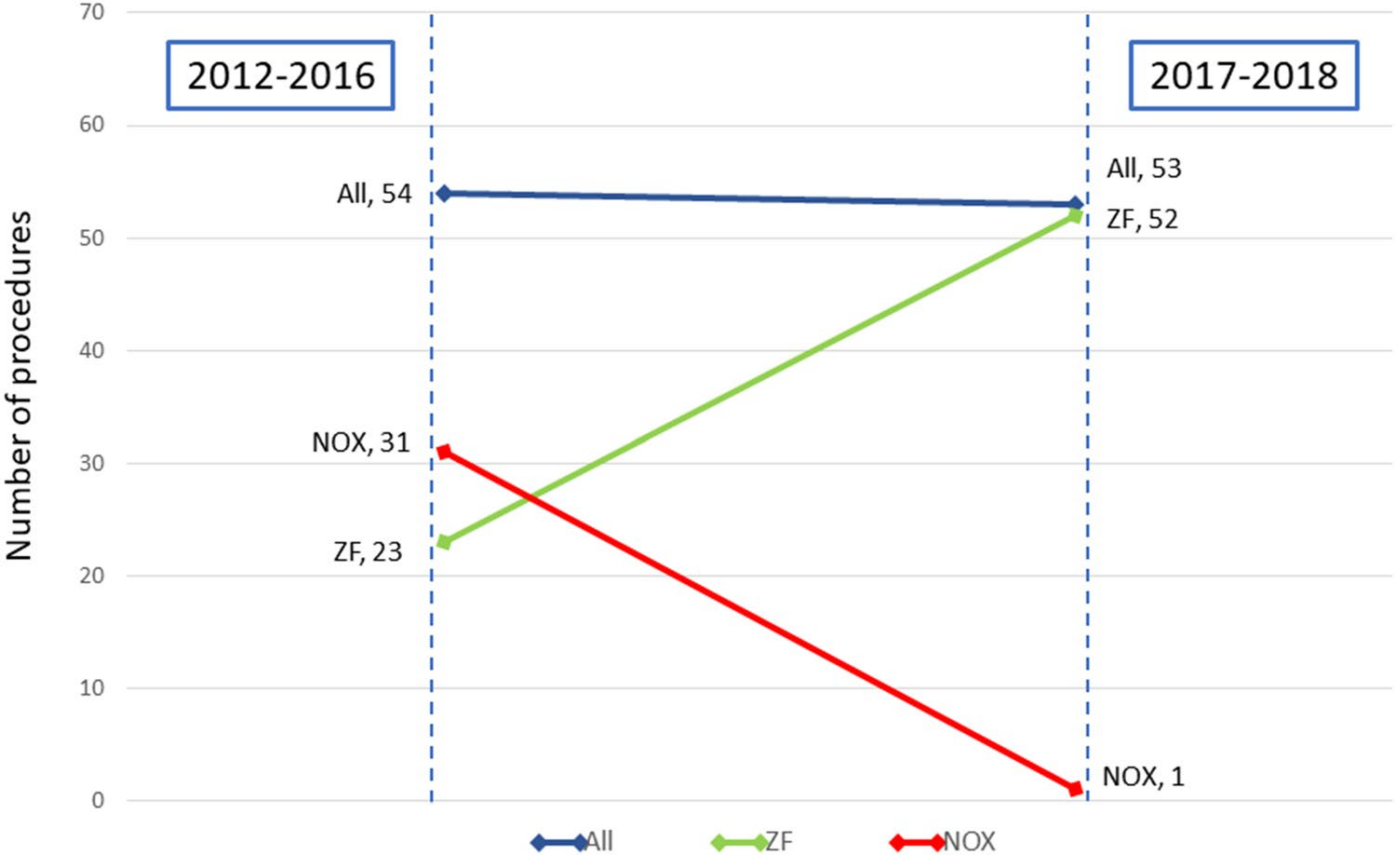
Difference in the preference for choosing zero over near-zero fluoroscopy between the early (2012–2016) and late study period (2017–2018). The increased preference of the operator for choosing the ZF procedure in the late study period is significantly higher. *NOX* near-zero fluoroscopy, *ZF* zero fluoroscopy

**Fig. 5:**
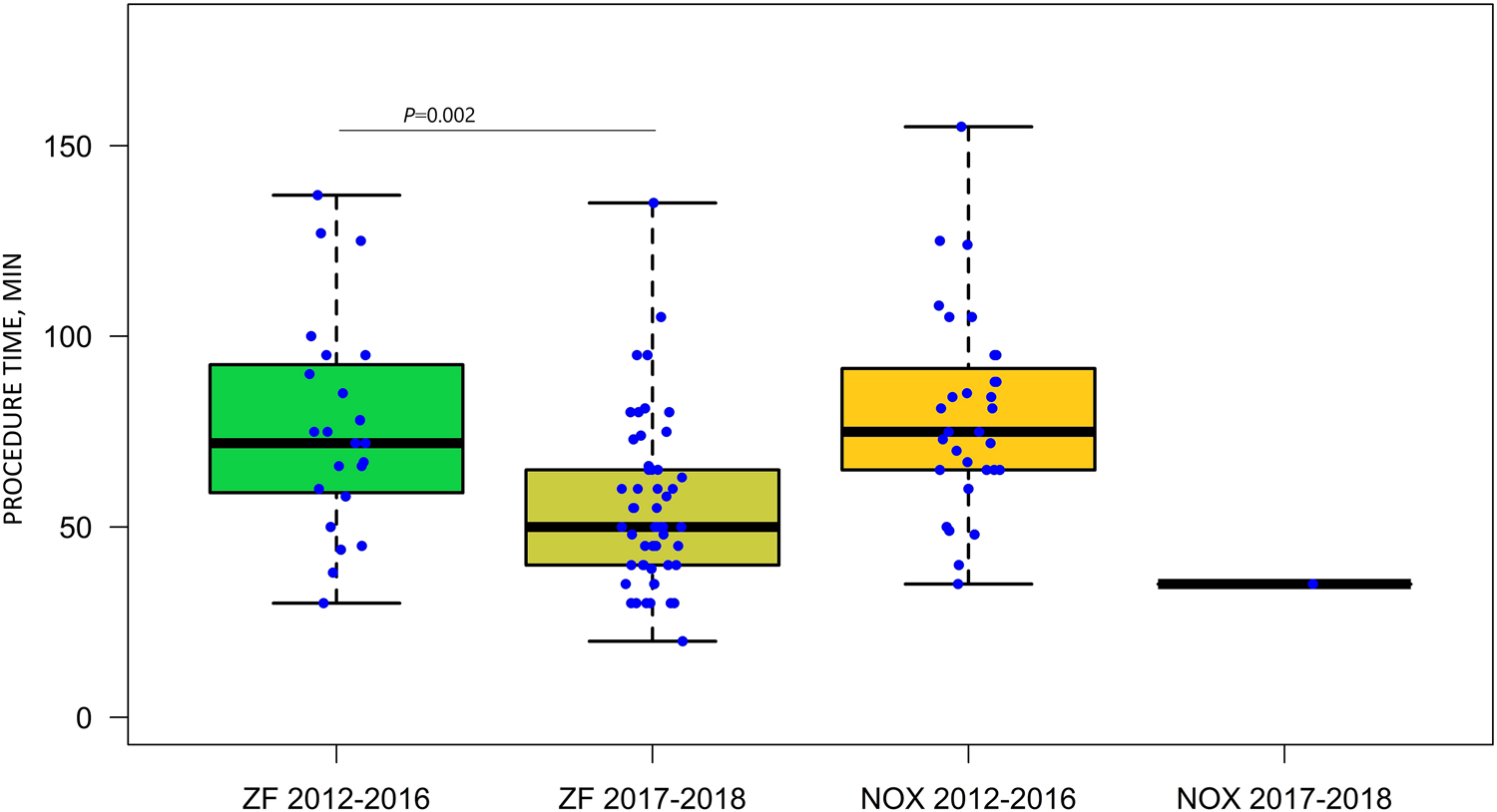
Procedure duration depending on ablation approach and the study period. Boxes and whiskers represent medians and interquartile ranges. For ZF approach the median procedure time, comparing the early and late period, significantly decreased in the latter. *NOX* near-zero fluoroscopy, *ZF* zero fluoroscopy

Three equally experienced operators and 3 middle-advanced EP fellows performed ablation procedures in 10 sites using the ZF or NOX approach. With growing experience, the preference for using the ZF approach has significantly increased over the recent years. Stratification by the period of ablation (early: 2012–2016 and late: 2017–2018) revealed that ablation with the ZF approach was performed in 23 of the 54 cases (43%) in the early period, as compared with 52 of the 53 cases (98%) in the late period (Fig. 4). Moreover, the analysis by period of ablation showed that the median procedure time decreased significantly over time for the ZF approach (Fig. 5).

**Fig. 6:**
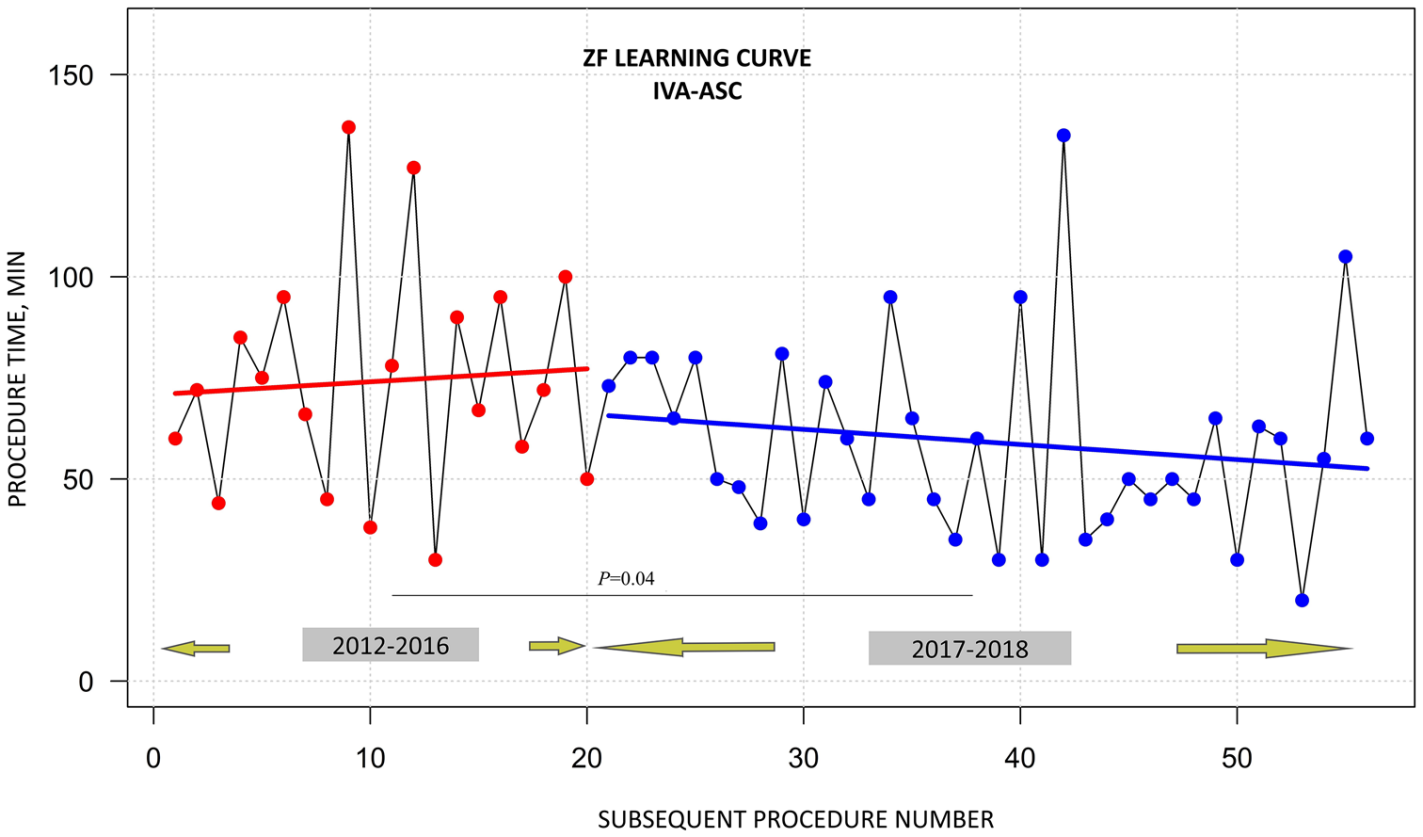
Learning curve for the zero fluoroscopy approach in ablation of idiopathic ventricular arrhythmia from the aortic sinus cusp obtained for a single operator over the study period (explanations in the text). *ASC* aortic sinus cusp, *IVA* idiopathic ventricular arrhythmia, *ZF* zero-fluoroscopy approach

The learning curve was also assessed for the ZF procedures performed by a single operator (56 procedures, which constituted 52% of all ZF and NOX procedures for IVA-ASC). The results are presented in Fig. 6. The procedure time in the late ablation period was shorter by a median of about 15 min than in the early period along with the increasing number of performed RFA procedures. The median procedure time for this operator in the years 2012–2016 was 72.0 [range 45.0–80.0] min vs 57.5 [range 43.8–73.2] min in the years 2017–2018 (*P* = 0.04).

The outcomes of the ZF and NOX approaches depending on the ablation periods are shown in Fig. 3. Generally, ablation outcomes in the late period [in which ZF procedures predominated (98%), with only 1 NOX procedure] were comparable to those in the early period when the ZF approach was used in the minority of cases (42%). The overall success rates in the early compared with late periods were as follows: periprocedural, 93% vs 100% (*P* = 0.12); 1-month, 87% vs 85% (*P* = 0.99); and 12-month, 85% vs 85% (*P* = 1.00).

## Discussion

### Main findings

This is the first retrospective analysis of multicenter standardized registry with at least 12-month follow-up demonstrating that RFA with the ZF approach may be safe and successful in the majority of patients with IVA-ASC when performed by an experienced operator. The study revealed the successful implementation of ZF approach and importance of appropriate training that resulted in an increased preference of the operator for choosing the ZF procedure over the studied time period, with no change in ablation outcome or the rate of complications. Moreover, the learning curve for the ZF approach demonstrated a significant reduction in the procedure time along with the increasing number of performed RFA procedures, which could be explained by the increasing operator’s experience as well as the use of electroanatomic and direct mapping of the predicted ablation site by computer software.

### Radiation exposure and other risks of coronary angiography

Over the last years, there has been a significant progress in the field of cardiac electrophysiology, with a rapid development of techniques that allow us to perform more complex and longer procedures on the one hand and to reduce the radiation exposure for medical staff and patients on the other. The cumulative effect of X-ray exposure is a serious problem. Even low doses may be potentially harmful, particularly for children, young adults, and pregnant women, but also for other patients, considering the total radiation exposure they have while undergoing modern tests and therapies throughout their lifetime [8, 9, 15]. There is also a significant risk for operators because protective lead clothing only partially reduces the harmful effects of radiation and may also cause serious orthopedic problems and fatigue [10].

Coronary angiography, although recommended to confirm a safe distance from the coronary ostia when ablation pertains the aortic root [1, 4], may be associated with significant risks and adverse events, including not only radiation exposure but also additional vascular injury, allergic reactions, coronary dissection, or renal insufficiency. Our registry data show that a careful use of electroanatomic mapping helps avoid fluoroscopy and angiography, at least in some cases, and helps perform the procedure safely.

### Available data

There is an increasing body of evidence showing that the use of the 3D-EAM systems significantly reduces fluoroscopy exposure without affecting the procedure safety and success rate in patients undergoing RFA for supraventricular arrhythmias and IVAs [8, 10, 11, 14, 16–25], including also data from our center [26]. However, at present, there is almost no evidence on the use of RFA with the ZF approach in patients with IVA originating from the ASC. Zhu et al. [13] investigated a small population of patients (n = 23) undergoing RFA due to idiopathic PVC from the ASC. Zero-fluoroscopy ablation was performed with the CARTO 3 mapping system and showed a shorter procedure time and a similar success rate in comparison with patients who underwent conventional fluoroscopy ablation. In another study on a group of 32 patients referred for RFA of IVA-ASC, Hoffmayer et al. [12] demonstrated the efficacy of 3D-EAM systems (NavX system, St. Jude Medical and CARTO system, Biosense Webster) without the need for coronary angiography although with the support of intracardiac echocardiography. The periprocedural success rate during 1-month follow-up was 83%, which was similar to the rate observed in our study. However, except from coronary angiography, no data concerning other use of fluoroscopy were provided.

To our knowledge, our study is the first multicenter analysis to confirm not only the feasibility but also long-term efficacy and safety of the ZF approach in RFA of idiopathic PVC/VT from the ASC without the use of intracardiac or transesophageal echocardiography. It also demonstrated the learning curve for the ZF approach to ablation in patients with IVA-ASC throughout the 6-year period, with a significant increase in the preference for choosing the ZF technique and a decrease in the procedure time. At the same time, the overall periprocedural and long-term procedural success rates in our registry were comparable to outcomes reported in other studies using ablation with classic fluoroscopy for IVA-ASC [1, 13].

### Advantages of the zero-fluoroscopy approach and future implications

Electrophysiologists and medical staff largely underestimate the risks associated with radiation exposure. Recent data have shown that most experienced electrophysiologists have an annual exposure of about 5 mSv, which is twofold or threefold higher when compared with diagnostic radiologists, with a cumulative lifetime attributable risk of 1 cancer per 100 exposed individuals [20]. An equally important issue is the protection of patients. Picano et al. [20] demonstrated that patients could achieve a cumulative effective dose of 100 mSv after 4 RFA procedures plus 2 or 3 computed tomography scans, which causes an additional risk of cancer in 1 per 100 cases.

The multicenter randomized NO-PARTY trial (Near Zero Fluoroscopic Exposure During Catheter Ablation of Supraventricular Arrhythmias) revealed that the patient’s risk of cancer incidence and mortality when using the minimal fluoroscopic approach was reduced by 96% compared with conventional procedures, with a parallel reduction in the years of life lost and the years of life affected [19]. Given such an overwhelming advantage in cancer prevention, it would be reasonable to increase the awareness of the negative effects of radiation exposure among electrophysiologists and patients, to promote ZF and NOX procedures, and to include these procedures as part of every educational and training program for electrophysiologists [19]. Our study may significantly contribute to resolving the problems of radiation exposure, as it demonstrated that only after 4 years since the introduction of the ZF approach, it has become a more preferable option than the NOX approach, with a comparable success and safety profile.

The development in invasive, non-fluoroscopic 3D-mapping systems, other non-invasive imaging techniques (e.g. cardiac magnetic resonance) as well as physicians’ experience enable implementation of ZF catheter ablation in ASC. The quality of data gathered from 3D-EAM resulted in rare use of virtual maps merging with 3D chambers shells (ex. left atrium) from preprocedural acquisitions. Therefore, even in aortic root, ZF mapping of arteries ostia is feasible and safe after appropriate training. Similar approaches are used to validate ZF anatomy for transeptal puncture, implantation of intracardiac leads for cardiac resynchronization therapy via coronary sinus and performing hybrid procedures without use of fluoroscopy.

### Limitations

Our study has several limitations. First of all, it was a nonrandomized study but retrospective observational registry. The choice between the ZF and NOX approach was at the discretion of the first operator depending on his or her preference. However, we believe that this does not affect our results because one of our findings was the increased preference for choosing the ZF approach. Moreover, some patients were switched from the ZF to NOX approach during the procedure. However, we documented the reasons for the switch, and these patients were included in the ZF group in an intention-to-treat analysis.

Another limitation is the small number of patients. However, to our best knowledge, this is one of the largest multicenter studies on RFA procedures using the ZF or NOX approach in patients with IVA-ASC. The study by Zhu et al. [13] compared the ZF approach with conventional ablation. We believe that our experience supports the use of the ZF approach in patients with IVA-ASC, particularly in young individuals and those at higher radiation risk. However, these results should not be generalized to patients with structural heart disease and PVC/VT from the ASC in whom coronary angiography might be obligatory or recommended.

Finally, in some patients with IVA or other arrhythmias, the substrate represents atrioventricular connections or site of origin is located close to the ASC. Therefore some patients require application from bipolar sources, the coronary venous system or fluoroscopically validated coronary arteries to assess the distance from an ablation catheter to even small epicardial coronary arteries [27, 28]. Therefore, it may not be possible to omit coronary angiography in every patient with PVC/VT originating from the left ventricular summit or arrhythmia substrate (PVC/VT foci, atrial tachycardia, atrio-ventricular connections) originating from the ASC. Moreover, additional studies and parameters (simulations, auditing, training programs) are needed for the widespread use of ZF approach, especially in ASC arrhythmia origin.

## Conclusion

Radiofrequency ablation with zero fluoroscopy can be completed by experienced operators in the majority of patients with IVA originating from the ASC. After appropriate training and electroanatomic mapping of the aortic root, the ZF approach became a more preferred ablation technique for IVA-ASC than the NOX approach. We showed that ablation of IVA-ASC with the ZF approach guided by the 3D-EAM system has similar feasibility, safety, and effectiveness to the NOX approach.

## Supplementary Information

Below is the link to the electronic supplementary material.

Supplementary file1 (DOCX 13 KB)
