# Supplementary material for: Implementation of zero or near-zero fluoroscopy catheter ablation for idiopathic ventricular arrhythmia originating from the aortic sinus cusp
Source: Int J Cardiovasc Imaging. 2021 Oct 28;38(3):497–506. doi: 10.1007/s10554-021-02432-8 (PMC8927012; doi:10.1007/s10554-021-02432-8)
Supplement: Supplementary file 1 — Supplementary file1 (DOCX 13 KB) [file 10554_2021_2432_MOESM1_ESM.docx]

Supplementary Table S1

Major complications of ablation procedures:

1. Valvular damage

2. Tamponade

3. Myocardial ischemia/myocardial infarction

4. Thromboembolism

5. Heart block

6. Vascular access complications

7. Any other complication requiring procedural interventions

Minor complications:

1. Any complications other than those listed above
